# Anterior Spinal Fusion for Thoraco-Lumbar Idiopathic Scoliosis Comparing Less Invasive Concave versus Traditional Convex Approach: A Pilot Study

**DOI:** 10.3390/jcm13154383

**Published:** 2024-07-26

**Authors:** Glenn Buttermann

**Affiliations:** Midwest Spine & Brain Institute, 1950 Northwestern Avenue, Stillwater, MN 55082, USA; gbuttermann@midwestspine.net; Tel.: +1-(651)-430-3800; Fax: +1-(651)-430-3827

**Keywords:** anterior spinal fusion, concave, convex, idiopathic scoliosis, lateral fusion, Oswestry Disability Index, outcomes, Visual Analog Scale

## Abstract

**Background/Objectives**: Anterior spinal fusion for primary thoracolumbar or lumbar (TL/L) adolescent idiopathic scoliosis, AIS, has advantages over posterior fusion, particularly in saving motion segments below the fusion construct. Traditionally, the approach is anterolaterally from the convexity. In adult degenerative scoliosis, the lateral or anterolateral approach may be performed from the traditional or from the concave approach which is less invasive and gives comparable outcomes. The purpose of the present pilot study was to assess the feasibility of the less invasive concave approach for younger AIS patients and compare it to the traditional convex approach over a 5-year follow-up period. **Methods**: The two cohorts were assessed by comparing pre- to postoperative radiographs, and clinical outcomes for pain, function, self-perception of appearance, and opinion of surgical success were prospectively obtained. **Results:** Radiographs found that primary TL/L scoliosis significantly improved from 53° to 18° (65%) for both the concave and convex cohorts. Sagittal alignments remained stable and there was no difference between cohorts. Coronal balance improved in both cohorts and sagittal balance was stable for both. Clinically, VAS back pain improved significantly for both cohorts initially and remained improved in the concave group. Leg pain, pain drawing, ODI disability, and VAS appearance scores improved and there was no difference between cohorts. The self-rating of success of the procedure was 100% at early and late follow-up periods. There were no neurological/surgical complications. **Conclusions:** The concave approach for anterior fusion for TL/L AIS is feasible with comparable radiographic and clinical outcomes to the traditional approach.

## 1. Introduction

Progressive or advanced primary thoracolumbar or lumbar (TL/L) adolescent idiopathic scoliosis, AIS (Lenke type V curve), may be treated surgically with a fusion. The approach types can vary. Common approaches for flexible curves are anterior spinal fusion, ASF, or posterior spinal fusion, PSF. Traditionally, ASF is from a convex approach which is anterolateral or lateral (usually anterior to the psoas muscle). This approach may be performed from the right or left side, that is, ASF may also be from a concave approach [1]. For rigid curves, a combined anterior and posterior, ASF/PSF, approach with osteotomies may be used.

There is controversy regarding the best approach for flexible TL/L curves for AIS, as each approach has advantages and disadvantages. This holds true for both adolescent and adult patients depending upon how much degenerative disc disease, stiffness, bone density, or stenosis they may have concurrently. PSF approaches may be less painful and avoid the risk of “thoracotomy syndrome” (lower costal neuralgia) but may have a greater incidence of proximal junctional kyphosis due to the disruption of the posterior musculature [2,3,4,5,6]. Unlike the ASF approach, PSF is familiar to all spine surgeons.

Comparative studies have suggested that a traditional ASF can save the number of levels required for fusion and thus would be considered motion-sparing [4,5,7,8,9,10]. The levels not fused, or saved, by the anterior fusion technique are often below the fusion construct and may lower the chance of long-term distal adjacent level symptomatic degeneration. Usually, ASF does not end below L3, which is favourable [8,11]. Longer distal constructs, particularly if they extend to L4, which is more common in PSF, may result in greater distal disc degeneration and a decrease in activities [8,12,13]. ASF may require the assistance of an access surgeon if the spine surgeon is not familiar with the ASF approach.

The approach for ASF can be on a traditional convexity; however, often this entails a more extensile approach and a possible take-down of the diaphragm. However, with a concave approach, one can often use a semi-minimally invasive approach, which may also spare the diaphragm and reduce convalescence. The comparison between concave and convex ASF approach has received very limited study in the past. Although, to the author’s knowledge, there are no known studies of concave ASF for younger AIS patients, a few comparative studies exist in patients in the scoliosis subset of adult spinal deformity, ADS, of which most also used posterior instrumentation, or ASF/PSF [14,15]. These ASD studies concluded that improvements in radiographic and clinical outcomes were similar regardless of whether the ASF approach was from the concave or convex aspect of the lumbar curve.

The purpose of the current pilot study is to investigate the feasibility of the concave approach to ASF for TL/L AIS, and to compare the concave to the traditional convex approach in regard to radiographic and clinical outcomes, particularly for AIS patients with relatively flexible curves.

## 2. Materials and Methods

### 2.1. Study Cohort Description

This is a comparative retrospective study of 2 cohorts of consecutive Lenke Type 5 AIS curves who had a minimum follow-up of 5 years, all of whom had prospectively collected data. The early cohort all had a traditional convex approach. Upon refining the minimally invasive concave technique in mild/moderately severe adult degenerative scoliosis (ADS) cases, the author then used this technique (modified from transpsoas to anterior to the psoas) for AIS patients, which is the recent cohort [16,17]. Patients and/or parents consented to chart information abstraction prior to surgery. After Institutional Review Board review, the analysis included a review of radiographic and clinical outcomes of adolescents, as well as adults with adolescent-onset idiopathic scoliosis. 

### 2.2. Radiographic Analysis

Radiographic outcomes compared standing X-rays for preoperative primary TL/L curvature in both coronal and sagittal planes to postoperative imaging. Preoperative supine lateral bending X-rays were obtained to assess curve flexibility. Additionally, the compensatory thoracic, T, and lumbosacral, LS, scoliosis, and biplanar spinal balance changes were assessed and compared between preoperative and postoperative films.

### 2.3. Surgical Technique

**Figure 1:**
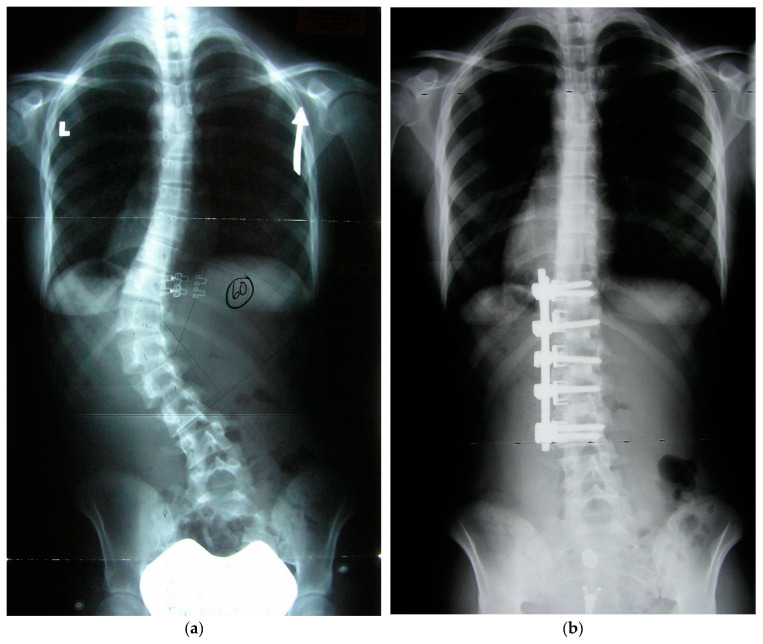

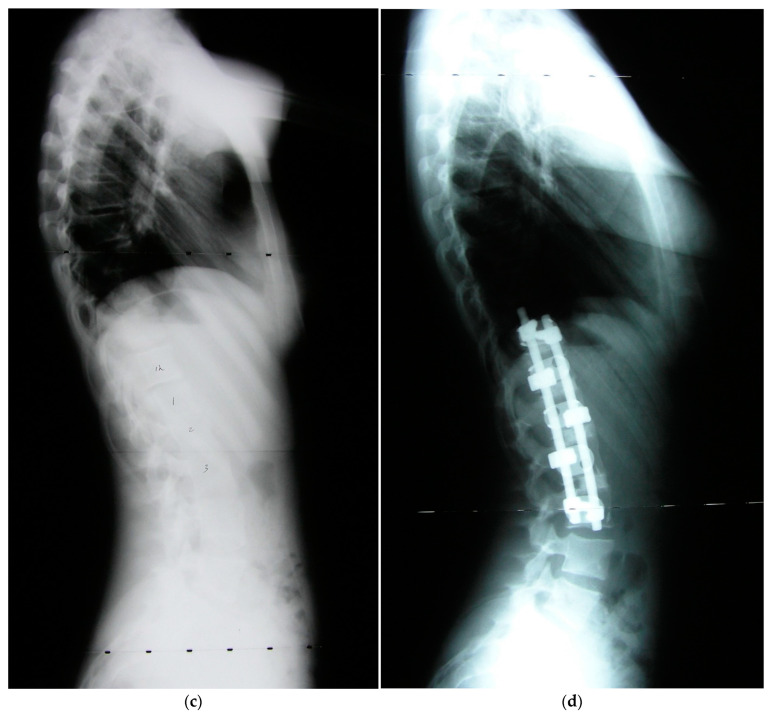
Preoperative coronal and sagittal radiographs (**a**,**c**) and postoperative radiographs (**b**,**d**). (**a**) AIS Type 5 preoperative coronal (upright indicated by arrow) radiograph with 60 degrees scoliosis. (**b**) Postoperative radiograph at one year after traditional convex technique.

**Figure 2:**
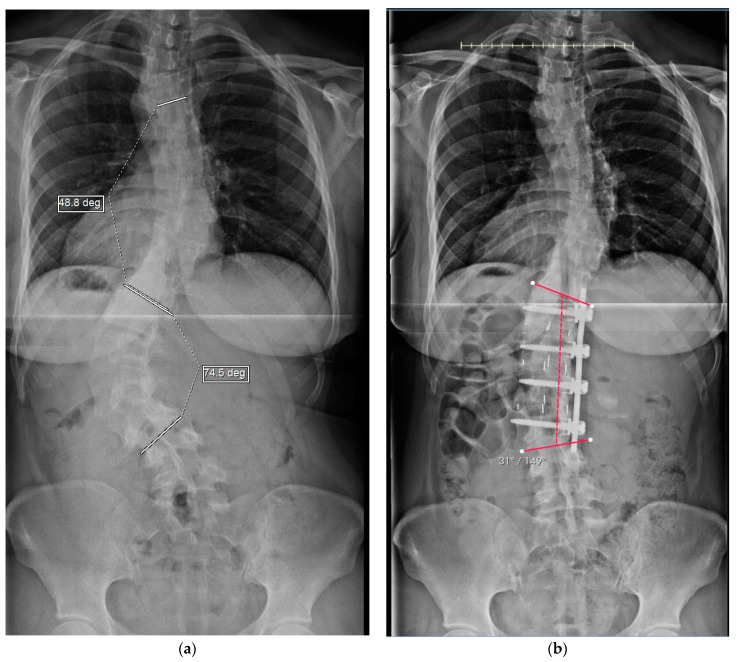
Preoperative and postoperative coronal radiographs (**a**,**b**).

**Figure 3:**
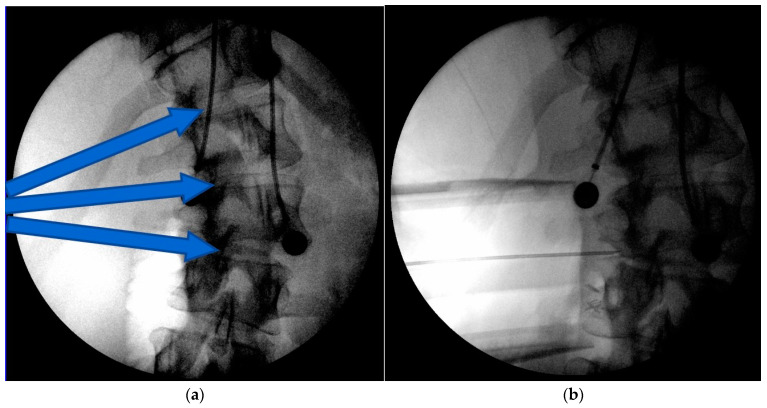

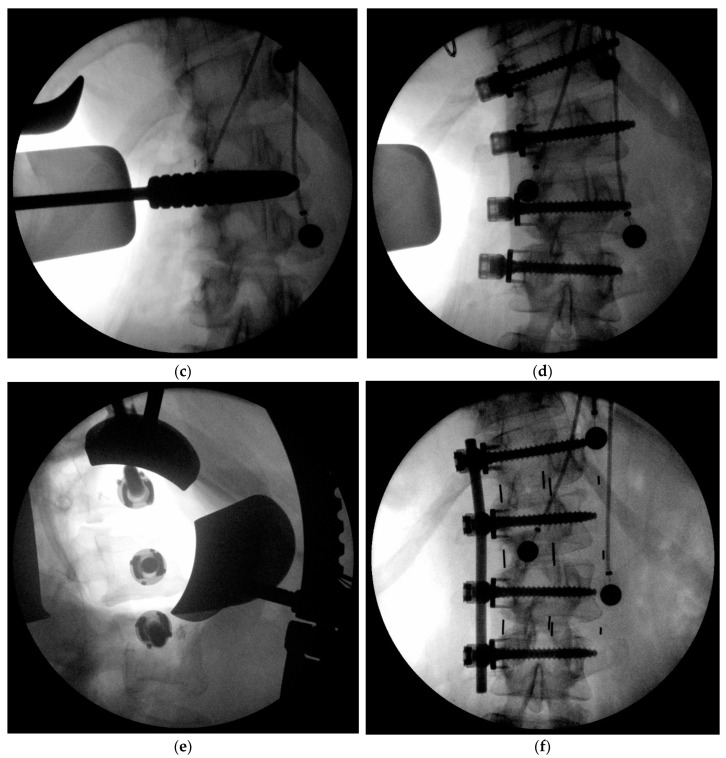
Intraoperative images of the less invasive technique to the concave apex of the curve (**a**), The arrows indicate the planned trajectories and location for the wound incision. A radiographic marker is placed to confirm levels (**b**). Coronally tapered trials are used to determine interbody implant sizes starting at the end vertebral levels and working subsequently to the apical levels (**c**). The screw staple implants are then placed at the end vertebra first and then subsequently placed through the apical vertebral levels (**d**,**e**). Distraction is then applied between each segment and the rod secured to the transverse vertebral body screws (**f**).

The traditional surgical convex approach is well known for ASF (Figure 1). The concave surgical approach is not as well known and is briefly described (Figure 2). The anesthetized patient is positioned in a lateral decubitus position with the curve convexity down and the concavity up. The bed is flat during this portion of the procedure, yet the table’s flexion axis is at the apex of the patient’s curve to allow for subsequent table bending to assist in curve correction (below). After prepping and draping, imaging is obtained to plan the less invasive incision over the concave apex of the curve (Figure 3a). Because the spine is rotated away, the actual incision may be slightly anterior from the true lateral. In thin younger patients, one can use a minimally invasive approach using a 3-bladed minimally invasive retractor. The minimally invasive technique is used in 7/11 cases. A standard retroperitoneal approach is made. The wound may be deep and thus if the patient is large, a more extensive open approach will be needed. A radiographic marker is placed to confirm levels (Figure 3b), and the segmental vessels are ligated after approaching the lateral spine just anterior to the psoas. Psoas muscles are released from the antero-lateral aspect of the underlying discs and vertebral bodies and then retracted posteriorly. The most proximal disc and distal disc segments are then verified for access. Typically, the upper disc is approached first, most commonly just below the diaphragm. A large annulotomy and a generous discectomy is performed, the cartilaginous endplates removed, and the bony vertebral endplates prepared. A coronally tapered interbody device trial is used. After sizing, the transvertebral body screws (the author prefers mono type screws) and staples are placed transversely across the vertebral body parallel to the endplate and obtaining bicortical purchase. The OR table may be flexed as needed to facilitate the placement of the trials or screw/staple constructs. Subsequently, the distal disc level is approached, and typically, a combined coronally tapered and lordotic trial is used distally. This is repeated subsequently at the apical levels (Figure 3c). The screw staple implants are then placed at the end vertebra first and then subsequently placed through the apical vertebral levels (Figure 3d,e). Staples are used to prevent toggle of the screws, which are predominantly in cancellous vertebral body bone. 

After the endplates have been prepared and the transverse screws and staples have been placed, the coronally tapered interbody cages with or without lordosis combined with bone graft are placed working from the end intervertebral levels toward the apex. At this point, flexing the table and placing the coronally tapered implants achieves approximately 2/3 of the desired scoliosis correction as well as achieving lordosis. Spinal rotation usually spontaneously corrects due to the ligamentotaxis of the PLL and the release of the concave annulus. The appropriate length rod may be contoured in a slight lordosis if necessary and then placed into the screw heads. Manual retractors are preferred during this part of the procedure in order to expeditiously instrument the rod to the screws over multiple levels. Distraction is then applied between each segment and the rod provisionally secured with set screws to the transverse vertebral body screws (Figure 3f). This distraction maneuver, which may be repeated, accounts for an additional 1/3 of scoliosis correction. A final tightening of the set screws completes the instrumentation portion of the procedure.

### 2.4. Clinical Analysis

Clinically, prospective outcomes were obtained pre- and postoperatively (minimum 5-year follow-up period) and consisted of back and leg VAS pain and appearance VAS scores (Visual analog score: 0 to 10 scale with 0 = no pain or normal appearance and 10 = extreme pain or severely deformed/“crooked”). Outcomes also measured pain drawing (distribution of pain over the body surface with 0 = no painful areas) and loss of function ODI-9 (Oswestry Disability Index: 0 to 100 scale with 0 = no disability) [18,19,20,21,22]. Pain medication use was tracked and categorized as either nonnarcotic or narcotic. Additionally, patients completed a “yes/no” self-assessment of success, whether they would have the procedure again under similar circumstances, and whether they would recommend the procedure to others. In the adult patients, stenosis, degenerative disc disease, and disc herniations were tracked, but not specifically treated.

### 2.5. Statistical Methods

Statistical comparisons were made between the two groups, initially using a two-sample *t*-test for all the outcome scales [23]. For each *t*-test, a Shapiro–Wilk test was performed to assess normality due to the small sample size. If the null hypothesis failed to be rejected, the data were assumed to be normal. If the null hypothesis was rejected, there was sufficient evidence that the data were not normally distributed, and a non-parametric Mann–Whitney U test (Wilcoxon signed rank test) was performed to take the place of the *t*-test. A Mann–Whitney U test was also utilized if the assumption of homogeneity of variance was violated. All statistical analyses were carried out using Citrix SAS 9.4 TS Level 1M6. *p*-values less than 0.05 were considered significant.

## 3. Results

### 3.1. Demographic Findings

The two cohorts were similar in that most patients were female. Both groups had predominantly young adults and adolescent patients; however, both groups also had one patient each older than 50 years of age. The traditional convex approach cohort were slightly younger (mean age = 21 years old; median = 17; range 13 to 52) compared to the minimally invasive concave cohort (mean age = 28; median = 21; range 13 to 62) (Table 1). One patient in each cohort was lost to follow-up at 3–4 years postoperatively.

### 3.2. Radiographic Findings

Preoperative radiographic findings (Table 1) found similar mean preoperative scoliotic curves of the lumbar (thoracolumbar) regions measuring 53° for both the concave and for the traditional convex groups (Table 1; the significance column represents the *p*-value between the two cohorts). Postoperative curves were a mean of 18° and 19° for the concave and convex groups, respectively, which was predicted by their preoperative bending radiographs. The mean change in both groups was 34° (65% improvement). Compensatory thoracic and lumbosacral curves improved uniformly. The coronal radiographic improvements (TL/L primary, and compensatory T and LS scoliosis) were all significant for both groups (*p* < 0.02). It is also notable that the median number of levels fused in the concave group was three, whereas in the convex group it was four. Additionally, the LIV was lower, on average by ½ level, for the traditional cohort. Preoperative coronal imbalance was similar in both groups. Postoperatively, the coronal decompensation was reduced to 0.5 cm and 1.2 cm in the concave and convex groups, respectively. Sagittal parameters remained stable. Between the two cohorts, there was no significant difference in these preoperative and postoperative radiographic parameters. Four patients had notable distal junctional disc degeneration of which two were fused to below L3, and one had a residual deformity of the lumbosacral curve of 17°. 

### 3.3. Clinical Findings

Clinical outcomes found that most patients had preoperative back pain, particularly older patients. Only four patients in total of both groups combined, had a preoperative back pain VAS score of less than 3 of 10. Preoperative pain VAS scores had a moderate correlation with age (r = 0.61) and were insignificantly worse for the concave compared to the convex group. The comparison of the pre- and postoperative pain scores over a minimum 5-year follow-up period found improvement (Table 2). Both groups had improvement in pain in the early postoperative period (*p* < 0.03) and remained significantly improved over the 5-year follow-up for the concave cohort (*p* < 0.001 at all follow-up periods). However, the change in pain scores relative to preoperative scores was significantly greater for the concave group relative to the convex group (*p* < 0.05 for all follow-up periods greater than 1 year). ODI disability scores were worse for the concave cohort preoperatively (*p* = 0.034). Postoperatively, ODI improved and remained improved similarly for both cohorts and was significant for the concave cohort (*p* < 0.025 for all follow-up periods after the first year). ODI improvements were significantly better for the concave relative to the convex group (*p* < 0.03 for all follow-up periods greater than 1 year). Preoperative pain drawing scores were significantly worse for the concave compared to the convex group (6.6 ± 4.1 vs. 3.3 ± 1.8, *p* = 0.024). Postoperatively, pain drawing scores were improved and similar between the groups. The VAS deformity scores were significantly improved (*p* < 0.0001 for both cohorts), and this was sustained over the entire follow-up period. In summary, given the small sample size in each cohort, which allows for significant false positive or false negative findings, the improved back pain scores favoured the concave cohort at the 2-, 3-, and >4-year follow-up periods (*p* = 0.4). There was no significant difference in leg pain scores, pain drawing scores, disability scores, or deformity scores between the two cohorts at any of the follow-up periods. Readers are advised as to the low power of the study due to the small sample size.

Preoperatively, 7/11 and 1/11 concave patients used non-narcotics and opioid medications for pain, respectively. Preoperatively, in the convex group, three out of eight and two out of eight used non-narcotics and opioid pain medications, respectively. At final follow-up, two patients in each group used non-narcotics and one adult patient in each group used opioids. Regarding their surgery, patient self-assessed success was 100% for both groups at all follow-up periods. This was also true for their recommendation for the procedure to other scoliosis patients for all periods. However, at the early follow-up period, 80% to 88% indicated that they would have the surgery again under similar circumstances and this declined to 63% at 5 years in the convex cohort.

### 3.4. Perioperative Findings

There were no neurological or surgical complications in either group, and no postoperative complications, including no cases of post-thoracotomy neuralgia/pain syndrome and no pseudoarthrosis in either group. However, transient numbness in the ipsilateral anterior thigh was noted in three patients in the concave cohort. Intraoperative mean estimated blood loss, EBL, was insignificantly greater in the concave group, and the mean length of stay, LOS, was 4 days for both cohorts. Two patients noted above had additional surgery for fusion extension for distal adjacent segment disc degeneration with lateral-listhesis instability, one at 3.6 years in the concave and one at 9.3 years in the convex group, after their index ASF for scoliosis. 

## 4. Discussion

The current pilot study demonstrates that ASF by a less invasive concave approach for Type 5 AIS is feasible and may be as successful as the traditional convex procedure. There were no significant differences in radiographic results, and clinical outcomes between the two surgical ASF approach cohorts may favour the concave approach. The lower number of motion segments fused favoured the concave cohort too. 

Perioperatively, the EBL of the current study was less than that reported in a prior review and consistent with prior reports of ASF for Lenke 5 AIS [3,5,9]. LOS, an average of 4 days, was similar for both cohorts and was less or within the range of prior reports summarized in a recent review [5].

The radiographic improvement of the present study, for the primary TL/L curve, as well as for the compensatory T and LS curves, was comparable between the two cohorts of the present study. Furthermore, it was comparable or favourable relative to prior reports of ASF [3,5,8,9,24,25,26,27]. Coronal spinal balance improved and was similar to prior reports [8,26]. Sagittal radiographic parameters did not change significantly and were similar to these prior reports too. Distal adjacent degeneration was observed in our patients regardless of approach and was predictable with the risk factors previously described of residual deformity and fusion below L3 [11,12]. 

The percentage improvement of the global primary coronal curve in prior studies may also be related to the number of levels fused. A recent review of ASF vs. PSF compared the number of levels fused [5]. Of nine comparative studies, six found that ASF had fewer fusion levels, typically four to five levels, which was one to two levels fewer than PSF patients. The review also found three comparative studies in which the number of levels was the same for ASF and PSF at five to six levels for both on average. Surprisingly, the more levels fused did not result in greater curve correction. The patients in this present small study had only three to four levels fused for their ASF (favouring the concave approach) and had approximately the same amount of curve correction as reported in the review article for both traditional ASF and PSF techniques. The differences between the current study concave approach vs. that reported for the PSF approach suggest the possibility that a concave annulus and soft-tissue release allows for greater correction per intervertebral segment. 

**Figure 4:**
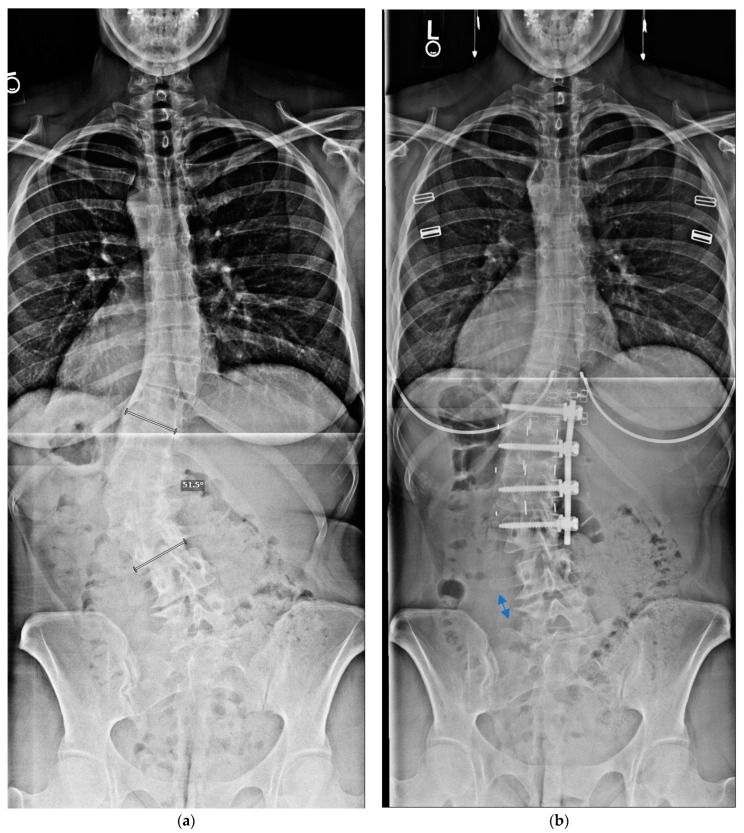
Preoperative and postoperative coronal radiographs (**a**,**b**) of a patient who had a resolution of left L5 pain symptoms after surgery. This demonstrates increased opening, indicated by the arrows, in the left L4–5 disc height in the fractional lumbosacral curve below the corrected T12–L3 fusion construct.

Maximizing the number of caudal levels not fused is thought to be advantageous, not only in terms of sparing mobility, but also in minimizing adjacent segment disc degeneration below the fusion construct [11,12]. Prior reports have suggested that anterior and/or lateral fusions can reduce the number of motion segments treated relative to PSF. Additionally, realigning the spine favourably improves the biomechanical loading of the adjacent lower discs [28]. The present study supports that contention. Most of our patients had their fusion at L2 or L3 as the lowest instrumented vertebral, LIV, level. Our few patients with a lower LIV or greater residual lumbosacral, LS, curvature had distal degeneration, with two having additional surgery. Interestingly, two of the adult AIS patients had a resolution of significant leg pain; one had foraminal stenosis and the other a posterolateral disc herniation extending into the foramina, both at the level below the fusion construct and on the concavity of the LS fractional curve. Upon correction of the spinal deformity, which secondarily corrected the LS curve, the disc spaces and angulation were neutralized (Figure 4a,b), and radiographs suggested that the foramina opened on the concavity of the fractional curve below the primary curve, which presumably resulted in their resolution of preoperative leg symptoms.

Clinically, back pain improved significantly at the initial follow-up period. The back pain improvement was maintained over the minimum 5-year follow-up period of this study for the concave approach cohort. The current study found that the concave group had greater back pain reduction, and this difference was just over the threshold of MCID [29]. The greater pain reduction in the concave cohort may reflect the greater preoperative pain that this group had, which is due to their older age with more degenerative findings. The reader is to be reminded that the number of subjects is small and that there is a potential for false negative interpretation (statistical Type II error) when comparing the results of groups. A power analysis based on the pilot data of this study, to achieve a power of 0.8 for all the parameters of this study, was determined to be approximately 25 patients in each group. Leg pain resolved and disability improved. Pain and ODI reduction with ASF has been noted previously [26,30]. Many prior studies of ASF used the SRS outcome questionnaire. However, this outcome instrument may be difficult to interpret and usually the individual domains are reported. The SRS questionnaire’s Pain domain is often, but not always, improved in prior studies of ASF [5]. Satisfaction/success results were high in this small study and favourable compared to a prior mixed AIS surgery study [31].

Studies using an anterior only concave approach for Lenke 5 scoliosis fusion have not been previously reported in AIS, to the author’s knowledge, let alone a comparison of concave vs. convex approach. Comparing the current study of types of surgical approach for ASF in AIS patients to others is therefore limited to those of adult spinal deformity, ASD. These scoliosis patients within the ASD studies typically had less severe curves; however, they may be less flexible [16,17,32]. These ASD studies also usually found that optimal results were achieved if ASF was combined with posterior instrumented fusion. The reports that used anterior instrumentation used small plates rather than the traditional transvertebral body screws connected to a lateral rod which allows for distraction/compression between the screw-heads. In the ASD literature, there are only a few comparative reports of the concave vs. convex approach [14,15]. These ASD studies found no differences in radiographic or clinical outcomes or in complications, similar to the present AIS study. Transient ipsilateral thigh numbness in the present study is similar to that noted in the ASD studies [32].

The present study has advantages in that no patients were lost to follow-up; however, there were significant limitations. These were the non-randomized retrospective nature of the study, as well as the small sample size in each group which can lead to type-2 statistical errors, that is, the study is underpowered and would need to have two to three times as many subjects to have sufficient power to address all the changes in outcomes that were assessed in this study. Therefore, there is the possibility that the improved outcomes of the concave group could be even better than what is described in the present study. In addition, clinical outcomes are primarily driven by pain. Pain is multifactorial and cannot be entirely explained by the data collected in this study which focused on deformity and less on disc or facet joint degeneration, which may also be a source of pain.

The clinical implications of the present study suggest that a concave approach for Lenke 5 AIS, which uses a minimally invasive method and a laterally placed rod that allows for additional curve correction in addition to the interbody coronally tapered cages, may give superior outcomes. This would be a preferred option if larger studies support the findings of this pilot study. Looking into the future, treatments of Lenke 5 AIS will continue to evolve. Anterior vertebral body tethering is being explored. Although this technique still markedly reduces motion, it is not a “fusion” which is attractive to patients. Currently, tethering has challenges; studies of short- and midterm-length follow-up periods have found approximately 20–30% failure rates of the tethers and less correction than fusion studies [33]. Furthermore, revision surgeries and additional treatments for the distal junctional level may be more frequent. Possibly, a hybrid surgery will be a solution with a minimally invasive concave approach for ASF at the apex over one to two levels and a non-fusion stabilization of the end segments.

## 5. Conclusions

The concave approach results in substantial radiographic improvement similar to the traditional convex approach for ASF in thoracolumbar and lumbar, Lenke Type 5, AIS, and is also applicable to adults with adolescent-onset AIS and relatively flexible curves. Solid fusion is predictable but, just as in any ASF, proper endplate preparation is required. Although clinical outcomes in the present study favoured the concave approach group, there are important limitations in this pilot study, particularly the very small sample size, which question the outcomes favouring the concave approach. Therefore, future larger studies will need to confirm or disprove our preliminary findings.

## Figures and Tables

**Table 1:** Group demographics, surgical parameters and radiographic comparisons.

	Concave (n = 11)			Convex (n = 8)			Significance
Median Age (years)	21.8	±	16.3	17.0	±	12.7	NS
Female (%)	91			88			NS
# Motion Segments Fused	3.4	±	0.5	4.0	±	0.0	*p* < 0.001
Estimated Blood Loss (ml)	325.0	±	378.2	462.5	±	143.3	NS
Length of Stay (days)	4.3	±	1.8	3.9	±	0.6	NS
Lowest Level Fused (LIV)		5 × L2			2 × L2		
		4 × L3			5 × L3		
		2 × L4			0 × L4		
		0 × L5			1 × L5		
**Coronal Radiographic Results (Mean ± SD)**							
Preop TL/L Curve (degrees)	53.1	±	11.2	52.5	±	9.6	NS
Preop Bending (degrees)	13.9	±	14.5	19.5	±	12.4	NS
Post-op Curve (degrees)	18.4	±	10.9	18.6	±	12.4	NS
Scoliosis Change (degrees)	−34.7	±	9.3	−33.9	±	11.3	NS
Scoliosis Improvement	65%	±	19%	65%	±	68%	NS
Preop T Curve (degrees)	26.5	±	9.9	32.0	±	11.9	NS
Post-op Curve (degrees)	16.8	±	9.6	18.1	±	12.4	NS
Scoliosis Improvement	37%	±	21%	52%	±	36%	NS
Preop LS Curve (degrees)	26.5	±	6.3	27.4	±	9.3	NS
Post-op Curve (degrees)	7.5	±	3.1	7.6	±	5.1	NS
Scoliosis Improvement	72%	±	11%	71%	±	15%	NS
Preop Coronal Decomp (cm)	2.4	±	1.4	2.7	±	1.4	NS
Post-op Coronal Decomp (cm)	0.5	±	0.6	1.2	±	1.0	NS
**Sagittal Radiographic Results (Mean ± SD)**							
Preop T4-12 Kyphosis (degrees)	38.1	±	9.0	27.6	±	18.8	NS
Post-op Curve (degrees)	37.2	±	5.7	29.1	±	15.0	NS
Kyphosis Change (degrees)	−0.9	±	6.8	1.6	±	7.3	NS
Preop T12-S1 Lordosis (degrees)	45.6	±	17.7	39.5	±	10.1	NS
Post-op Curve (degrees)	48.8	±	14.2	38.0	±	10.7	NS
Lordosis Change (degrees)	3.2	±	10.7	−1.5	±	2.4	NS
Preop T10-L2 Kyphosis (degrees)	13.6	±	13.4	4.4	±	7.4	NS
Post-op Curve (degrees)	8.7	±	9.5	3.3	±	3.4	NS
Kyphosis Change (degrees)	−4.9	±	10.3	−1.1	±	7.0	NS
Preop Sagittal Decomp (cm)	−1.4	±	1.5	−1.6	±	1.5	NS
Post-op Sagittal Decomp (cm)	−1.3	±	1.9	−1.1	±	1.6	NS

TL = Thoracolumbar, T = Thoracic, LS = Lumbosacral, NS = Not Significant.

**Table 2:** Clinical results (mean ± SD).

	Concave (n = 11)	Convex (n = 8)	Significance
Preop Back Pain VAS	6.1 ± 3.1	4.2 ± 2.1	*p* = 0.024
7–12-month Post-op Pain VAS	2.4 ± 2.3	2.0 ± 2.0	NS
1–2 yr Post-op Pain VAS	1.1 ± 1.4	2.9 ± 2.3	*p* = 0.024
2–3 yr Post-op Pain VAS	1.9 ± 1.7	3.0 ± 2.4	NS
4–6 yr Post-op Pain VAS	1.4 ± 1.1	3.0 ± 2.4	NS
Leg Pain VAS	2.0 ± 3.5	1.4 ± 2.0	NS
7–12-month Post-op Pain VAS	0.5 ± 0.9	0.5 ± 0.7	NS
1–2 yr Post-op Pain VAS	0.0 ± 0.0	1.3 ± 2.7	NS
2–3 yr Post-op Pain VAS	1.1 ± 1.8	0.9 ± 1.0	NS
4–6 yr Post-op Pain VAS	0.1 ± 0.3	0.1 ± 0.4	NS
Preop ODI	34.3 ± 18.5	16.3 ± 13.9	*p* = 0.034
7–12-month Post-op ODI	18.5 ± 19.1	10.6 ± 10.0	NS
1–2 yr Post-op ODI	13.0 ± 8.4	10.0 ± 16.9	NS
2–3 yr Post-op ODI	10.7 ± 9.9	10.6 ± 10.0	NS
4–6 yr Post-op ODI	6.0 ± 7.7	8.0 ± 13.4	NS
Preop Deformity VAS	7.0 ± 2.1	5.7 ± 1.1	NS
7–12-month post-op Deformity VAS	1.7 ± 2.0	1.4 ± 1.3	NS
1–2 yr post-op Deformity VAS	1.3 ± 0.9	1.5 ± 1.7	NS
2–3 yr post-op Deformity VAS	1.2 ± 0.8	0.5 ± 2.3	NS
4–6 yr post-op Deformity VAS	1.0 ± 1.3	1.6 ± 2.1	NS
Self-assessment of Success at 1 and 5 yrs	100%, 100%	100%, 100%	NS
Would Have the Procedure Again	80%, 100%	88%, 63%	NS
Would Recommend the Procedure to Others	100%, 100%	100%, 100%	NS
Additional surgery	1	1	

VAS = Visual Analog Scale; ODI = Oswestry Disability Index.

## Data Availability

Data are contained within the article.
